# DIY HIV prevention: Formative qualitative research with men who have sex with men who source PrEP outside of clinical trials

**DOI:** 10.1371/journal.pone.0202830

**Published:** 2018-08-23

**Authors:** Sara Paparini, Will Nutland, Tim Rhodes, Vinh-Kim Nguyen, Jane Anderson

**Affiliations:** 1 Department of Anthropology and Sociology of Development, Graduate Institute of International and Development Studies, Geneva, Switzerland; 2 Department of Social and Environmental Health Research, Faculty of Public Health Policy, London School of Hygiene and Tropical Medicine, London, United Kingdom; 3 Homerton University Hospital NHS Foundation Trust, London, United Kingdom; University of Toronto, CANADA

## Abstract

Pre-exposure prophylaxis (PrEP) with antiretroviral medication is an effective, evidence-based option for HIV prevention. In England, issues of cost-effectiveness and of responsibility for commissioning prevention services have so far led National Health Service (NHS) England to decide not to commission PrEP. Given the significant lag between the awareness of PrEP efficacy and the opportunity to obtain PrEP through traditional health care routes, many gay and other men who have sex with men (MSM) have turned to ‘DIY PrEP’, purchasing generic formulations of PrEP for themselves on the internet or via other alternative routes. However, there is very little research on DIY PrEP practices and no qualitative study with DIY PrEP users in the UK. A formative qualitative study was conducted in 2017 to inform the development of an intervention (PrEP Club) to support DIY PrEP users and improve the safety and experience of this prevention strategy. Focus groups were held with 20 MSM who are based in London and are obtaining PrEP through means other than clinical trials, to explore their accounts of sourcing and using PrEP and the experiential meanings of these. In this article, we report findings from this first, formative study and present the different practices involved in finding out about PrEP, buying it and ascertaining legitimacy of sellers and products. We reflect on the uncertainties participants described related to actually using PrEP, including deciding on drug dosing and monitoring their health. Finally, we present the results of the discussions participants had about the kind of support they had received, the help they would have liked, and their views on proposed interventions to support DIY PrEP users, such as PrEP Club.

## Introduction

The data accrued from multiple clinical trials over the past decade demonstrate pre-exposure prophylaxis (PrEP) with antiretroviral (ARV) medication to be an effective, evidence-based option for HIV prevention [1–3]. There has been an exponential growth in the visibility of PrEP, as well as advocacy for its use, particularly amongst gay and other men who have sex with men (MSM) and trans women, following the high-profile efficacy findings of the iPrEx trial (in 2010) in the US, Brazil, South Africa and Thailand [1], the PROUD trial (in 2015) in England [2], and the IPERGAY trial (in 2015) in France [3]. Internationally, countries such as France, Australia, Canada and the US have implemented different PrEP programmes, and international health efforts are directed towards PrEP interventions for a wider range of populations globally [4].

However, in the United Kingdom (UK) the real-world availability of PrEP remains fragmented. The impact of the confirmed clinical efficacy of PrEP in research studies has been operationalised differently in health policy environments across the four nations of the UK. As of November 2017, National Health Service (NHS) Scotland commissions PrEP through sexual health clinics using generic formulations, NHS Wales is implementing PrEP through a public health trial with additional monitoring, whilst the situation in Northern Ireland remains unclear due to the currently uncertain broader political situation in their Assembly. In England, issues of cost-effectiveness and the legal responsibility for the commissioning of prevention services and technologies were at the heart of NHS England’s decision in 2015 not to commission PrEP after the end of the PROUD trial [5]. At the end of 2017, however, NHS England too has started a new large PrEP implementation trial (called IMPACT), with a view to answer questions not addressed in the PROUD trial due to its design and pilot size.

Willingness to use PrEP and its acceptability, particularly amongst MSM, have been previously evidenced in the UK. In a 2011 online survey of 1259 MSM in England [6], awareness of PrEP was generally low (80% of reported having been unaware of PrEP), but when asked whether they might use PrEP if available, around half of men who had not tested HIV positive (52.4%) reported they would consider using PrEP if prescribed at a sexual health clinic. In a 2011 cross-sectional survey of 842 HIV-negative men (recruited in London gay venues) around half reported they would consider taking PrEP [7]. In a sexual health clinic survey of 121 HIV-negative MSM in a Manchester sexual health clinic, over a third stated they would be ‘very willing’ to take PrEP [8].

In a cross-sectional survey of 17 gay commercial venues in Edinburgh and Glasgow [9], around half of the 1393 men included in the analysis reported they would consider taking PrEP daily. In a mixed method study on understanding PrEP acceptability in Scotland, almost half of 929 MSM in a cross-sectional survey reported they would be likely to use PrEP should it be available. HIV negative or untested men in focus groups in the same study shared concerns about major side effects [10]. Findings from further focus groups and in-depth interviews in Scotland with MSM and African participants, including HIV positive, HIV negative and untested individuals [11], show that understanding of effectiveness and of adherence were viewed as barriers to PrEP uptake, with self- perception of being at low-risk for HIV meaning few participants saw the benefits of PrEP.

Since this earlier research, awareness of PrEP across the UK has been heightened by the vocal engagement of community activists, health promoters, PROUD trial researchers and sexual health clinicians, along with controversy in the media about NHS England’s stance on its provision since 2015 [12]. Many MSM in London have thus known that PrEP is an effective intervention to prevent acquisition of HIV for a number of years. Yet for some time they were only able to obtain PrEP drugs via a private clinic if willing to pay approximately 400 GBP per month.

The England–wide NHS England PrEP IMPACT trial began recruiting in October 2017, aiming to enrol as many as 10,000 patients. Eligibility criteria include: gender, sexuality and sexual behaviour; HIV status and testing history; partner’s HIV status and viral load; and self-reported, anticipated use of condoms. Trial places are being taken up quickly (the trial website shows almost half of all places had been assigned by the end of February 2018), with differences geographically and across clinics.

Given the significant lag between the publishing of evidence of PrEP efficacy and the inability to obtain PrEP through traditional health care routes, many MSM have, in recent years, turned to the internet to buy generic PrEP for themselves, or to friends and acquaintances to obtain PrEP through alternative means. Yet, while widely reported through social and other media [13–14], there has been an absence of research investigating men’s use and experience of self-sourced—or what may be called “DIY”—PrEP.

We therefore undertook a qualitative study in England with MSM who self-source PrEP through means other than clinical trials. This formative study describes practices of sourcing and using DIY PrEP adopted by a group of London-based MSM, and explores experiences associated with DIY PrEP for these men. This research begins to identify the needs of people buying PrEP outside of formal settings and provides a basis for further research and formative evaluation, as a step towards the development of interventions by key stakeholders, commissioners of services, clinicians and community-based health promoters wishing to support DIY PrEP users. The study also opens up questions for further social science research into DIY practices in HIV prevention and health more broadly.

## Background

PrEP is a prevention strategy that involves an HIV negative person using HIV antiretroviral medication (usually a combination of tenofovir and emtricitabine) to prevent the acquisition of HIV. The branded single tablet combination preparation Truvada was approved for prevention by the US Food and Drug Administration in 2012, and its use has increased throughout the world, especially amongst other MSM [15]. Due to the aforementioned issues with access, in the UK, increasing numbers of MSM legally access PrEP online and purchase much cheaper generic versions of the medication manufactured in India and Thailand via online pharmacies (at around 40 GBP per month).

The emergence of websites such as *PrEPster* and *IWantPrEPNow* (hereafter: *IWPN*) have signposted people to relatively reliable sources of generic PrEP, linking them directly with online pharmacies, explaining costs and procedures, and answering individual queries where possible. These websites were formally launched and linked in October 2015, both being established by un-funded grassroots activists, although sharing information about online availability of PrEP by the same activists who went on to establish *PrEPster* (one of whom is Author 2 in this article) had been going on prior to then. A number of key clinicians were instrumental in providing clinical support to DIY PrEP users, such as kidney monitoring and pre-PrEP use HIV tests. Two London clinics provided free therapeutic drug monitoring tests, enabling PrEP users to ascertain that they were using genuine preparations [16].

Sharp falls in new HIV diagnoses amongst MSM in some clinics in London have partially been attributed to DIY PrEP use [17]. Between October 2015 and September 2016 new diagnoses amongst MSM in London fell by 29%, and decreased by as much as 32% in five major sexual health clinics in the city compared to the same period in the previous year [17] with a dramatic drop of 80% in one London clinic since January 2015 [18]. Although accurately quantifying the exact contribution of PrEP to this drop in HIV infection is challenging, estimates suggest that around a third of prevented infections amongst MSM in London clinics in the relevant period may have been attributable to the use of PrEP, both within clinical trials and by online purchases [17].

Nonetheless, many self-purchasers of PrEP do so with minimal support, often navigating online sites that can be confusing and raise concerns about credibility of sellers and legitimacy of products. In addition, PrEP users might start the medication without the recommended clinical procedures and support, which, according to professional guidelines [19–20], should include 4th generation HIV tests, kidney function tests, and advice on different PrEP dosing regimens. This carries a number of risks, not least of reduced effectiveness of PrEP against HIV. It is therefore crucial that PrEP users receive as much support and information as possible. However, thus far, there exists little research on DIY PrEP practices, in the UK or internationally.

The extent of the demand for online PrEP remains unknown. Anecdotal evidence available to *PrEPster* and *IWPN* suggests that by October 2017 (when the IMPACT trial started) as many as 10,000 people may have been purchasing PrEP this way in the UK. This is an estimate number that was obtained from three months of sales figures extrapolated from one of the online pharmacy sellers, then doubled when an online survey ran by *PrEPster* in July 2017 showed that 50% of all respondents reported buying PrEP from this same seller. In terms of interest, between March 2017 and March 2018, the *PrEPster* ‘Buying Online’ webpage was viewed 100,000 times [21]. However, these indicative figures are in no way robust, and reliable evidence of the extent of DIY PrEP use remains unavailable at the time of writing.

Some details about ‘informal’ PrEP are reported in studies from Australia and France, from answers given to questions about the use of ARVs before condom-less sex contained in larger surveys with gay men [22–23]. These studies show a correlation between informal use of PrEP and, amongst other characteristics, younger age and high-risk sex practices (e.g. injecting drug use, group sex without condom). Clinical observation of internet-sourced PrEP (or ‘interPrEP’) in the UK has focused on issues of therapeutic drug monitoring and drug safety of generic formulations purchased abroad [16, 18], with one clinic also reporting observed trends in sexually transmitted infections amongst its approximately 640 generic (as a proxy for DIY) PrEP users [24].

However, despite significant media interest in DIY PrEP, little research describes and contextualises DIY practices and experiences. Much attention is currently going into monitoring and surveillance of the sexual behaviour and health of PrEP users over time [25–26]. Some have commented on anti-PrEP alarmism, or a moral panic about what happens to (particularly gay men’s) sex in the age of PrEP [27–29]. Qualitative studies of the perspectives and experiences of those who use, or may use, PrEP, have placed varying emphasis on reclaiming pleasure, demonstrating responsibility, reducing anxieties, eliminating the ‘sero-divide’ and other affective and material effects of PrEP [27–32]. Yet, with the exception of one article reporting on ethnographic research with gay men using informal PrEP in Paris [30], which focuses mainly on men’s motivations for seeking PrEP and the effect on their sexual lives, there is little evidence for how to develop support interventions for novel, DIY HIV prevention initiatives [33], and none from the UK.

In conjunction with the providers of sexual health clinical services and community-based organisations in England, the activist group *PrEPster* developed a proposal to pilot a peer-delivered intervention, ‘PrEP Club’. PrEP Club would strive to provide a comprehensive intervention informing people about current systems for obtaining and using PrEP in England, including DIY PrEP, and to assist them in navigating such processes. Additionally, PrEP Club would provide support and information on the associated clinical procedures needed when starting and whilst using PrEP, and answer common questions that early PrEP users might have (e.g. how soon PrEP works, different dosing regimens, or dealing with potential stigma linked to using PrEP).

To make sense to DIY PrEP users, it was essential that the design of PrEP Club was informed by formative evidence generated by current and former users of self-sourced PrEP (i.e. those NOT obtaining PrEP via a trial, nor through a formal medical prescription). In this paper, we report on qualitative research conducted in London as part of the preparation for PrEP Club.

## Design and methods

The primary aim of this formative qualitative research project was to understand the information and support needs of MSM who currently are, or who have recently, self-obtained and self-administered PrEP. Objectives of the study were to: explore the sources and dosing regimens of current and recent PrEP users; identify the key barriers and facilitators encountered by people when obtaining PrEP online; and understand the clinical, information and support needs of current and recent DIY PrEP users. The study also explored the acceptability of potential delivery models for a PrEP Club intervention by asking study participants to comment on proposed ideas for how it could be carried out.

Because of the small size of the study, the exploratory nature of the research topic, and the intention to ask PrEP users to share their views on the design of PrEP Club activities and interventions, focus group discussions were chosen as the method for data generation [34, pp.126-150]. Three focus groups of approximately 90 minutes each were hosted in a privately-hired central London location in the summer of 2017. Each focus group consisted of six or seven participants (total = 20 participants). A further three participants agreed to take part but did not attend the focus groups and no further reason was given. The number of participants reflects the formative and scoping nature of the study. Basic demographic characteristics of the sample are detailed in Table 1.

**Table 1:** Sample characteristics.

**Area of residence**	**N.**	**Education**	**N.**
Greater London	18	University/ College	20
Unknown	2	**Age**	
**Country of Birth**		29–34	6
UK	10	35–40	7
Italy	2	41–46	3
Philippines	1	46–51	0
Canada	1	52–56	2
US	2	Unknown	2
Ghana	2	**Gender**	
South Africa	1	Male	20
India	1	**Gender assigned at birth**	
**Ethnicity**		Male	20
White British	10	**Years living in London**	
White	1	<10	2
White European	2	10–20	6
White other	3	> 20	5
Other	1	Unknown	7
Black African	1		
Indian	2		

All focus groups were co-facilitated by Author 1 and Author 2, a male and a female researcher with long-standing experience in qualitative health research on sexual health and HIV. Aside from researchers and participants, no other person was present. Author 1 is conducting anthropological research on the topic of PrEP in England and Author 2 is a public health researcher and health promotion activist involved in community PrEP mobilisation. Both authors’ interests and objectives were explained to participants at the beginning of the focus groups.

### Recruitment and sampling

A purposive sampling strategy was employed. The study was open to MSM (including trans men) over 18 years of age who had self-purchased, and self-administered PrEP in the past 12 months; and MSM who had purchased and used PrEP in the past 12 months but were not currently using PrEP. For reasons of convenience the study was only open to men based in or around London. MSM were being selected as this population group is disproportionately impacted by HIV. In 2016, the prevalence of HIV amongst London MSM was 128 per 1000 compared to 57 per 1000 amongst MSM in the rest of England, and 54% of HIV diagnoses in England were amongst MSM [35]. Also, although there is no available national evidence on DIY PrEP use, MSM are likely to be the key population group obtaining and using (self-obtained and clinically-prescribed) PrEP in the UK [17, 36].

Participants were recruited through advertisements on social networking applications. From these advertisements, participants were directed to a website which had a participant information sheet as the landing page. This provided the email contact details of the research team members. Potential participants provided consent to be contacted by the researchers and gave preferences as to the best way for the team to do so.

### Consent and ethics

Participants first gave their consent to be contacted by the researchers and were given the opportunity to read the information about the focus groups ahead of taking part. Those who agreed to take part, were asked to give explicit informed consent. This required signature of a form which set out the details of the focus group process, data anonymisation procedures, confidentiality, analysis and use of findings. Consent to participate was given both verbally and in writing at the time of the focus group. A hard copy of the participant information sheet was given to all participants and any questions that arose were addressed by the research team before participants were asked to sign the consent form. Participants were given 30 GBP to compensate for their time and for travel expenses. Ethics approval was obtained from the Ethics Committee of the London School of Hygiene and Tropical Medicine.

### Data analysis

The topic guide used in the focus group discussions is shown in S1 Appendix. This was not made available to participants ahead of the discussion but topics to be covered during the focus groups were introduced at the beginning of each session. The topic guide was used to direct the flow of the discussion, rather than as a list of questions. The structure of the guide was also used to divide up the time for the focus group, which was set and maintained for participants’ convenience, into various areas of discussion. In these, the researchers aimed to find out about the practicalities involved in DIY PrEP and to prompt and give space to men’s own reflections and conversation with each other about their experiences. Descriptions of experiences and practices relating to DIY PrEP were gathered from researcher-led questions and participant-initiated accounts. Focus groups also allowed researcher interpretations to be tested and validated with participants.

The focus group discussions were audio recorded with participants’ consent. All transcripts and field notes were anonymised by the researchers, with any identifying characteristics removed. Transcripts were not returned to participants.

Data analysis proceeded in four different stages. First, interview recordings were summarized by each researcher and then exchanged for cross-checking against notes taken during the discussions. Secondly, using thematic content analysis [34, pp.209-218], each researcher coded participants’ answers to the set of questions about experiences of DIY PrEP as they emerged from the data, transcribed selected quotes verbatim, and identified key themes. Thirdly, two researchers exchanged their coding frame to reconcile different interpretations and applied the new coding frame to the summaries and quotes. Findings from this part of the analysis are presented in the Results section with regards to “sourcing” and “using” DIY PrEP. In a fourth and final stage, one researcher summarized answers given to the pre-identified set of questions about proposed characteristics of a potential PrEP Club intervention, to describe participants’ preferences in the Results section on “support”.

In reporting direct quotes in this article, we do not identify each participant. However, when summarizing and selecting verbatim quotes for this article, we have sought to report the direct words of as many different participants as possible.

## Results

In the results section that follows, we first give a brief overview of the different practices involved in finding out about PrEP, buying it and ascertaining legitimacy of sellers and products. We then reflect on the uncertainties men described related to actually using PrEP, including deciding on dosing and monitoring their health. Finally, we present the results of the discussions men had about the kind of support they had received, the help they would have liked, and their views on proposed interventions to support DIY PrEP users such as PrEP Club.

### Sourcing

#### Finding out about PrEP

There was a wide variety of ways in which participants first learned about PrEP and how to source it from websites such as *PrEPster* and *IWPN*. Many participants had read about PrEP in newspaper articles or online newsfeeds, such as on their Facebook pages. Around half were directed *to IWPN or PrEPster* by their clinicians when they attended sexual health clinics. This could be because they had repeatedly attended clinic “*after one too many PEP* (post-exposure prophylaxis) *events*”, and were thus identified by clinicians to be at a level of risk that may warrant the use of PrEP, or because they were “*over-testing*” or had had other discussions about their risk. In two cases, they had been offered to take part in the PROUD trial but had not been able to enrol in the study, and online PrEP was suggested as an alternative. Only one man in our study has reported to have bought branded Truvada through a private service available in his clinic, until he learned that he could purchase generic drugs online.

For earlier adopters attending the more PrEP ‘pioneering’ clinics, experiences of finding out depended also on the year when they were first introduced to PrEP. One man explained that at the beginning it “*certainly wasn’t 100% transparent*, *especially because it was over a year ago*. *I think it’s more out in the open*. *Now you go to* (central London clinic) *and they have billboard saying*: *you can buy PrEP online*”. Another early DIY PrEP user recounted how the doctor told him about PrEP and then wrote the name of the *IWPN* website “*on the back of an envelope*”.

For those who did not find out in the clinic, friends and sexual partners were key to getting information about PrEP—“*my friend was on it*. *I had a talk with him and that’s when I started my journey of education and learning*”–and also provided assistance in navigating its purchase online. Of note, four men had first heard of PrEP from friends or sexual partners when travelling to the US—*“you go to NYC and you are strange if you are not on PrEP and you are HIV negative*, *quite frankly”*–then came back to the UK and found out about *IWPN and PrEPster*. This likely reflects the earlier approval of PrEP in the US in 2012.

#### Buying online

Most participants bought their PrEP online from one of three pharmacy websites featured on *IWPN*. One man had originally sourced his PrEP from the HIV treatment his HIV-positive partner was taking, whilst another had bought his first PrEP through a private prescription and, subsequently, online. *IWPN*’s recent decision to feature a ‘preferred provider’ had led some men to choose that same pharmacy website as an option, although some men who had longer-standing relationships with other sellers did not see this as a reason to change provider.

Price of PrEP drugs was important for some participants, with some moving between sites if they offered ‘deals’. However, the usability, trust-worthiness (or lack thereof) of a site, and range of payment options all factored into men’s decisions, too: “*I want to give these people my money but they’re making it difficult*”. Indeed, websites offering the cheapest option made some men hesitate: “*if it’s the cheapest you are a bit suspicious but if it’s the second cheapest then it’s fine*.”

Very few participants encountered issues with the non-arrival of purchased PrEP although a minority of men described paying additional customs charges on their orders. Issues with PrEP not arriving when expected were generally due to stock-outs with some of the earlier online sellers, or because men had miscalculated how long a delivery would take. Customer service issues were raised as most sellers appeared to be operating with small numbers of staff, and were slow to respond to buyers’ questions: “*the whole system seems like it’s being run out of a garden shed*”.

#### Legitimacy

Dilemmas about legitimacy emerged in discussions about sources of information, online purchases, and the quality and safety of pills and manufacturing. There was agreement in all of the focus groups that help from some collaborating clinics (particularly the central London clinic where most participants had received PrEP-related care) was central to their decision to source PrEP online: *“the doctor pointed it* (the website) *out…nothing can be wrong with this”*. One man also went to a PrEP-related event attended by his clinician, in which *IWPN* website was mentioned and that “*gave it the seal of approval*”.

However, full endorsement of DIY PrEP by the NHS would have been “*preferred*, *rather than*, *you know*, *it’s your decision*”. Conversations with their clinicians about their current sex lives and condom use meant that some men felt a certain degree of urgency to take a new course of action. They found themselves in an unusual and interim place: being encouraged by clinicians to consider PrEP as an option, whilst being unable to obtain it from them. Hence, they took the final steps online and on their own.

For some men, there was a sense of normalisation as online purchasing was something that they did on a regular basis: “*adding it* (PrEP) *to your basket as if it’s a top from* (online clothing shop)”. Concerns about buying PrEP online at times were compared to those about buying any drug or irregular product—“*I get more nervous about buying poppers online”*. However, many others did not feel at all comfortable buying online, and mentioned that, at the beginning at least, it felt “*seedy”*, *“illicit” or “surreal”*.

The lack of certainty about the pills they were taking—“*something that comes from Hong Kong shipped from somewhere else*”—played on their mind and contributed to some of their hesitation in starting PrEP. Although a few had bought pharmaceuticals online before, such as painkillers or vitamins, they were not used to it: “*it is alien in this country to buy your own drugs*”. Moreover, some saw PrEP as a more serious product with potentially serious consequences: “*this is the one that can fuck up your life if it all goes wrong*.”

Ambivalence was mitigated over time as more peers started to use the websites, more medical staff recommended the sites, and websites seemed to become more ‘professional’. Initial lack of trust in the different PrEP sellers’ payments systems improved as transactions were carried out safely and products were received as planned.

### Using

#### Side effects

*How* to take PrEP, and the effect it would have on their bodies, were important questions with which participants experimented outside their comfort zone. Most men reported having been concerned about side-effects before starting PrEP. Three of the men mentioned having felt unwell after taking PEP in the past (or knowing someone who did), which caused them concern that PrEP would have the same effect. Although his case was by far the most extreme, one man stated that he waited for three years before starting PrEP, talking to the doctor every few months, until he gradually got enough information to “*put his mind at rest*”.

Five men reported experiencing some side-effects, however these had resolved within four weeks. Participants mentioned nausea, flatulence, and vivid dreams. None were severe enough to cause participants to stop the pills. Men were also able to compare their experience with those of their HIV positive friends: “*Truvada has been around for a long time and I have lots of undetectable* (HIV-positive) *friends and it’s part of their regimen*. *So*, *I guess I sourced my information from more than just the website*”. Hence, as side-effects did not manifest or quickly subsided, they were reassured that their concerns hadn’t been borne out.

#### Daily v on-demand

Almost all participants took PrEP on a daily basis, with four using on-demand, or ‘event-based’, dosing of PrEP. One man started with on-demand PrEP but switched to daily dosing because he struggled to establish how much PrEP to take. Of the men using on-demand dosing, one did so because it was *“a better financial option*. *It’d be a waste to take it every day*.*”* Another man, who also thought it was a cheaper method, used PrEP on-demand because he said he had such clear delineations between his sex life and social life that he was able to plan and forecast when sex would happen, and when PrEP would need to be used.

Nonetheless, the effectiveness of the drugs and their potential long-term toxicities remained as a worry: “*I take a break*. *It does play on my mind that I am taking a tablet every day*”. Whereas, as mentioned, some saw Truvada as an established drug, another man worried that its long-term effects are not really “*known*” as this medication has been around for “*about 20 years only*”. These concerns had to be weighed against men’s perceptions of sexual risk and what they saw as the uncertain effectiveness of on-demand PrEP.

For example, one man said he was taking PrEP daily in London, until he went to work in another city,

“*where the sex life isn’t quite as active*, *so I thought ‘this is pointless’*. *So*, *I started event-based* (PrEP) *and I thought I might stick to that*, *because one of my fears about PrEP is long-term damage and what that might be*. *But then I just found that I just felt safer taking it and being sure knowing that I had a steady dose*”.

This was echoed by another participant who found event-based dosage “*very*, *very nerve-wracking*”. He says he would worry about having taken the wrong amount, having miscalculated the hours, and “*inevitably* (he) *would add doses*”.

Some preferred daily PrEP because, whereas results about on-demand PrEP came from international trials (for example the French IPERGAY study) that have tested its efficacy, daily PrEP was the regimen tested by the English PROUD trial, and recommended by the NHS:

“*what if I have missed a pill*, *what if I slept in…is this OK*? *it’s the little questions*, *just having something authoritative and definitive that answers them*, *in one place that has been given that authority*. *Not just by the IPERGAY trial or by the authority in the USA*, *but by something that’s known*, *domestic and that I can point to for my own purposes*, *the medical profession here*”.

#### Monitoring and testing

Although much of the men’s own information came from a range of online and personal sources, it was almost invariably the discussion with their NHS sexual health clinicians that made participants decide to go ahead and buy PrEP. Subsequently, receiving therapeutic drug monitoring provided them with credible information because they trusted their NHS clinic. This finally gave legitimacy to their purchase (and consumption) of otherwise unknown pharmaceuticals, removing “*a lingering worry that I have a batch that’s dodgy*”.

Thus, for those who had been able to access these tests, therapeutic drug monitoring results were “*a huge reassurance*” in two ways: first, the tests confirmed that the generic PrEP drugs purchased online were ‘real’, something they could not do on their own: “*what if this is just a sugar pill*? *How can I assess what a good pharmaceutical website is*? *I can’t*. *I have no idea*”; secondly, the tests established that the drug was present in their bodies, which made them feel safe.

There was, however, confusion in all the focus groups as to the necessary kidney function tests, in particular with regards to the need for urine or blood samples, and to their recommended frequency, which appeared to vary depending on the healthcare worker advice in different clinics and on whether they were attending PrEP-specific care or not. Some men were not satisfied with the regularity of these tests if they were every six months, because they saw the monitoring as important to detect any harmful impact of the drugs: “*six or 12 months is a lot to wait to find out if there is any damage*”. In some cases, this led to men taking breaks from the pills.

### Support

#### Support when starting

Participants discussed what support they would have appreciated when first starting PrEP. As a minimum, a greater level of ‘endorsement’ by the NHS of PrEP products available online (for example in NHS branded educational materials) would have offered reassurance. Some form of vetting of the sellers by health officials would have offered further security instead of information “*coming from someone’s blog*”, such as from *IWPN*. A number of men said they would have also benefited from emotional support to assist them in the beginning of their PrEP journey: “*I felt like I was doing it on my own*.” Participants would have liked the information about PrEP and how to use it to be more conclusive and less ambiguous: “*the advice out there is all a bit woolly*. *It’s all ‘this should*, *this could’ there is no ‘this will’* “.

Almost every participant had accessed some level of clinical support from the NHS when using PrEP and talked-through the practicalities of using PrEP. There was widespread gratitude for the help received, and an understanding of the difficult situation in which their clinics found themselves given NHS England’s ambivalent stance on PrEP provision at the time when they started taking it. They commonly discussed how they saw central London clinics, in particular, as “*overwhelmed”* and *“overcome with demand”*.

At the same time, however, many participants articulated frustrations with the clinical support services offered, citing lack of consistent care or information, and health workers who were not always aware of the tests and procedures necessary for PrEP use. Some men also reported that they had been met with lack of knowledge and judgemental attitudes when raising their PrEP use with health care workers outside of sexual health clinics (e.g. in primary or emergency care).

#### Peer support

Beyond the clinic, friends living with HIV offered advice about taking the drugs. However, the most crucial support came from peers and partners who had used or were also using PrEP. This peer support element, through friendship and social media, was seen as an essential component of PrEP education by almost all participants, with peer experience being seen as “*less abstract*” and “*more trustworthy*” than a website: “*peers’ experience is important*, *especially if they’ve been taking it for a while*. *It saves you the trouble of following those dead-ends yourself*.”

Hence, when we introduced the notion of ‘PrEP Club’, as a peer-led intervention education and support intervention for DIY PrEP users, participants unanimously felt it would be welcome, needed and beneficial, and confirmed that such interventions would have been useful for them at the start of their PrEP journey. Views differed on the format and setting to conduct ‘PrEP club’: social settings they already attended were convenient but might not work for engaging with a health intervention; for some, ‘PrEP Club’ should be regular, fairly formal and offer on-going support, while others would prefer less intensive one-off events. A few commented on the need to take it out of the ‘mainstream gay scene’ to venues attended by more marginalised LGBT populations.

There was widespread agreement that, regardless of the setting, in-person, face-to-face interventions were preferable to online services. Although it was widely agreed that intervention events should include an opportunity for medical questions to be answered by appropriately qualified professionals, there was simultaneously a strong sense that interventions should be guided by the experiences of PrEP-using peers. This remained important for those who had navigated online purchase of PrEP—“*for now it’s crucial because of all the difficulties with buying stuff* (online) *so hearsay is not good enough*”—and to better understand the experiences of those who had used PrEP themselves—“*it’s better to hear ‘these are the side effects I personally experienced’ rather than ‘here’s a list of side effects’* “.

#### Support interventions online

Although many had accessed social media and other online information to find out, source and inform their DIY use of PrEP, there was ambiguity as to whether PrEP support interventions should be delivered online. When asked their views on the proposed concept of having a ‘PrEP user’ or ‘ambassador’ with an emoji who could be identified on online apps, this was seen as useful, but fraught with tensions and difficulties, commonly around how boundaries would be managed. Also, it seemed to be a repetition of what they saw was already happening in DIY PrEP (“*it takes only a few minutes to find someone who is using PrEP in London on an app as it is*”). They said they were already often offering such advice and support to peers, and that there was little added benefit to such a scheme.

A few men felt that an online ‘hook-up’ setting was inappropriate to undertake such an intervention (“*I don’t think people on those apps are looking for that kind of information*”), that they themselves would not want to be asked about PrEP as an app user (“*you don’t go onto Grindr to impart your knowledge … you go on for other reasons*”), and that social media companies may be in it for the wrong reasons (“*it’s only window-dressing for those companies who don’t really care about the gay community at all*”).

## Discussion and implications for intervention and social science research

The overall findings from this formative qualitative study indicate that DIY PrEP users are highly motivated “early adopters” who are keen and able to experiment with new strategies for HIV prevention, even in the context of partial information and the need for significant personal motivation. Yet the discussions also highlighted that participants depend on crucial peer support to obtain a prevention technology that was promoted through community engagement in clinical trials rather than as a public health intervention. This highlights the need for tailored interventions as well as further research and analysis to better understand how DIY PrEP users can be supported to ensure the best possible outcomes from these informal practices. After a discussion of the study limitations, the suggestions below begin to address the need for a more comprehensive DIY PrEP agenda.

The study has several limitations. First, this study was carried out by a team of researchers which included a prominent PrEP activist (Author 2), who was known to at least a few of the participants through his high visibility in the media and his involvement in the websites they had used–this was both a potential limitation and an asset for identifying and recruiting participants.

At the beginning of the study Author 2 described his activist role to all participants, and it was explained that critical views about the work of his activist group would be particularly welcome in helping with the design of PrEP Club. Focus group participants were a self-selected sample of vocal and engaged MSM who wanted to share their experiences to help with interventions that might help other men in their communities. It is possible that they would have withheld their views because of the researchers, but this is unlikely. Their accounts of their DIY practices so far were of satisfaction as well as of frustration, and they found fault with many of the ideas proposed with regards to PrEP Club.

The research team comprises social scientists, clinicians and activist all engaged in HIV prevention and with a diversity of opinion regarding PrEP. This was reflected in the iterative analysis and writing of this article.

Secondly, the study was limited as it was based on a small, convenience sample of London MSM who were recruited to explore ideas about PrEP Club or similar interventions. The sample of 20 men who participated in the focus groups is not representative of the MSM population of London and, due to the nature of these practices of self-sourcing, we lack reliable information about the characteristics of MSM buying PrEP online with which to compare our sample.

In particular, all our participants were highly educated with a university or college degree. The most recent available sample of informal PrEP users from France shows 68% of men were University/College educated, against 100% in our sample. In the Australian and French surveys [22–23] about or over a third of informal PrEP users were under 29 years of age, whereas everyone in our sample was over 29 years of age.

An additional limitation was that many relevant issues spontaneously raised in men’s discussion (for example, the impact of DIY PrEP on condom use) could not be further explored during the focus groups as they fell beyond the scope of the study and of the topics men had consented to contribute to.

The study was meant to better understand how to plan further support for DIY PrEP practices in England, with potential relevance to international contexts for informal PrEP, as feasible, rather to serve as an evidence base for rolling out PrEP interventions. Our data shows a need for concomitant peer-delivered information and education alongside specialist clinic support for DIY PrEP-users. The decision to start taking PrEP involved deliberation and discussion with many different actors, yet the endorsement of online, generic PrEP received in some clinics was particularly important in shaping men’s final decision to start PrEP. Although by the time of the focus group discussions many participants had settled into DIY PrEP use, when recounting their initial experiences, they clearly expressed that they would have preferred a PrEP service in their sexual health clinic to begin with.

Further, subsequent confusion as to the monitoring that is necessary and/or available has highlighted the difference between the experiences of those who had been able to access a PrEP specific service and the rest. It is likely that PrEP-related commissioning issues at the time in which participants had started using PrEP, and the novelty of caring for DIY PrEP patients, may have meant that sexual health professionals were working with significant uncertainty leading to inconsistency.

As PrEP use is scaled up, healthcare workers need to be supported in developing PrEP-specific skills to address the needs of PrEP users; this is already happening in Scotland through NHS PrEP services and in England and Wales through the learning gathered from public health trials. However, it is likely that healthcare workers may also have to continue to support a variety of PrEP users that includes those who source their drugs informally, and particularly patients who are accessing drugs online.

For example, although recent studies have confirmed the overall safety of ‘interprep’ formulations [16], PrEP users need confirmation of the authenticity of their prescription. The therapeutic drug monitoring available to some of the study participants has currently been discontinued as no ‘fake’ batches of drugs coming from the selected online pharmacies linked to *IWPN* and *PrEPster* have been identified [16]. However, if new sellers or manufacturer enter the market, it will be important to reconsider safety issues.

DIY PrEP users would also benefit from clear and standardised guidance on the clinical monitoring needed to assess long-term drug effects, and support around drug-drug interactions. Since individuals may access health services when they have already started PrEP by themselves, it will be important that the necessary monitoring is explained and put into place.

The focus group findings suggest that different kinds of peer-led interventions (short term; longer term; formal; informal) that educate and inform MSM (and other groups) about the possibility of DIY PrEP can be delivered in a variety of settings (clinical; social; pop-up visits to existing groups and clubs). Participants in this exploratory study clearly articulated different preferences with regards to mixing their social life with a what some saw as a more of an ‘health’ issue. This variety presents a challenge for health promotion with potential and current DIY PrEP users which warrants further research as well as evaluation of current practices.

Interventions aimed at supporting DIY PrEP might take into account findings that men would have liked–and would prefer–legitimisation of DIY PrEP from qualified medical personnel and from the personal experience of those who had been sourcing and using the same drugs. Health promotion planners might thus consider ensuring that peer-driven interventions are supported, as appropriate, by partnerships with clinical services.

In terms of research, although the social science of PrEP has made strides in recent years, findings from this formative qualitative study suggest that the experience of DIY PrEP raises its own set of questions. Firstly, there is a growing general tendency towards patient-centred ‘DIY’ intervention in the risk management of chronic health conditions, as well as for ‘home-based’ self-care [37], including in HIV health, such as via HIV home-sampling and self-testing initiatives [38–39]. DIY PrEP offers a case for exploring how patients manage the DIY organisation of their health care, including via internet-based treatments, and should thus be further explored.

Secondly, accessing PrEP outside of a clinical trial or service is akin to other forms of experimentation, more commonly analysed in relation to non-standard biomedical therapies and procedures that are unavailable due to price or to clinical guidelines. Like PrEP self-sourcing in England, these are often characterised by patient activism, online networks, and the involvement of actors across geographical sites in terms of, for example, drug production, logistics and purchase [40]. Some of these experimental practices (such as the ‘liberation procedure’ for MS patients) go against medical advice and consensus [41], others (such as ‘buyers’ clubs’ for HCV generic treatments) are instead supported by patient-clinician alliances even if they remain outside of health systems provision [42–43]. Studies related to these tangential practices would prove important to compare the context of DIY PrEP, in England and elsewhere. Research with health care providers is needed to illuminate what they need to deliver best care against the background of the hesitancy and confusion that DIY practices might engender, such as in the case of our study.

Thirdly, PrEP is unusual as it is not a therapy for an illness, but a prevention method. Keogh & Dodds [44] have already usefully outlined many of the related implications for social science, including questions of identity, intimacy and community, effect on institutions, economic considerations, and the need for appropriate evaluation of impact. However, at the time when their research agenda was introduced, the situation in England did not yet feature the current phenomenon of DIY PrEP.

As this exploratory study indicates, DIY PrEP generates its own issues of how clinically and epidemiologically supported understandings of personal levels of HIV risk are entwined with frustrations about lack of PrEP provision from the NHS. This takes DIY PrEP into a more radical domain of political practice and citizenship, which needs to be documented and analysed.

## Conclusion

This research provides an initial and exploratory understanding of the use of DIY PrEP within settings when PrEP is not formally available and where informal PrEP use has already been identified. The findings also bear relevance for settings where PrEP is formally available (such as France) or partially available (such as in Canada, the USA or Germany) but where ‘hybrid’ purchasing could be occurring. For example, individuals might resort to DIY PrEP when they do not have insurance coverage for PrEP (or cannot afford the premium) and are unable to access PrEP through that route, or where PrEP is available on a rationed/subsidised basis, but is not widely available. Understanding how to harness community activism and clinical partnerships in different contexts remains key to maximizing the HIV prevention impact of informal PrEP.

Our findings suggest that participants felt that the diffusion of information on how to access PrEP was initiated and sustained by their communities. NHS England’s decision not to commission PrEP as a routine service, meant that whilst they felt supported in the clinic *as patients*, they simultaneously felt neglected *as citizens*. DIY PrEP thus impacts care engagement for MSM in new ways and represents a significant shift from the use and expectations of publicly funded sexual health services so far.

Although a national PrEP trial is up and running, and an NHS-commissioned PrEP service may be envisaged at some stage in the future, we cannot be certain as to whether DIY PrEP sourcing and use in England will continue, grow or cease. Hence, this formative qualitative study, as the first to investigate this issue, offers valuable insight for practitioners and social scientists alike to begin to understand how best to support and contextualise DIY PrEP.

## Supporting information

S1 Appendix PrEP Club focus group topic guide. (PDF)
