# Supplementary material for: DIY HIV prevention: Formative qualitative research with men who have sex with men who source PrEP outside of clinical trials
Source: PLoS One. 2018 Aug 23;13(8):e0202830. doi: 10.1371/journal.pone.0202830 (PMC6107262; doi:10.1371/journal.pone.0202830)
Supplement: S1 Appendix — (PDF) [file pone.0202830.s001.pdf]

S1 APPENDIX. PrEP Club focus group Topic Guide.

This focus group is about your experiences of sourcing and using PrEP and how people might be supported in accessing and using PrEP.

PrEP use and ways of obtaining PrEP

How have you been using PrEP?

Prompts: Are you currently using; previously used; what dosing regimens are you using?

In what ways have you been getting hold of PrEP?
What website/s have you been using to find out about PrEP?
What website/s have you been using to obtain PrEP?
Are there other ways that people are obtaining PrEP (e.g. friends)?
How did you find out about these sites and sources?

Website sites and their use

The websites specifically (both information sites that link to purchase and the selling sites themselves).

What have been your considerations and issues about using and navigating PrEP websites?

Prompts:
Did you have any concerns about 'trusting' the websites?
What practical issues did you encounter with the sites?
What would have improved this use of the website?
Did the PrEP arrive - were there any issues (including VAT)?

Using PrEP

What have been the key considerations and issues when starting to use PrEP?

Prompts:
Did you know about any tests that could be done on the drugs you bought?
Did you personally get a therapeutic drug monitoring test done on your delivery?
Did you have any concerns about using PrEP?
Did you get any information from about using PrEP safely?
Did you have any medical support in using PrEP? If so, what did you get?
Did you experience any side-effects?

PrEP support you would have liked

Thinking about the three areas above (buying online; using PrEP; safety concerns):

What support and help would you have wanted when you first tried to obtain PrEP?
What support and help would you have wanted when you first started using PrEP?
What support and help would you want/have wanted when using PrEP on an on-going basis?
Who would you want/have wanted to provide this support and in what setting?

Prompts:
Clinicians? Peers? Online/offline. Face to face. Individual. Peer to peer? Group?

How important is it for other PrEP users to provide this information?

Considering development of interventions

PrEP Club is briefly described: a short face-to-face, group-based intervention that guides potential PrEP users through the processes of obtaining PrEP, highlighting the challenges and how to overcome them. The intervention assists in understanding the health support that is required when taking PrEP. Referral sources are provided.

If an intervention such as PrEP Club had been available at the time you started considering obtaining PrEP, would you have used it?

If you'd been able to speak to someone who had ever used or currently used PrEP (i.e. a 'peer mentor') would you have done so? This might, for example, include someone wearing a tee shirt or badge in a bar/club; or someone who is identified by a 'pin' on their online profile.

If you had the chance to receive a small package of training to be a PrEP 'peer mentor', is this something you would consider doing? What would be the key things you would want to be considered in developing such a project?

Any additional comments?

Thank you for your time.
